# Digestion in 1846

**Published:** 1846-12

**Authors:** 


*Digestion in* 1846.—At last the phenomena of digestion are enlightened: digestion is no more to be considered a simple but a complex function. There are as many digestions as organs. First, the stomach, by which animal food is dissolved; it is in carnivorous animals almost the only intestine and they require no other; their digestion is gastric; it is intestinal in herbivorous tribes. After the incisors and cuspidati come the molares: in the same manner after the carnivorous intestine we find the intestines which digest grains and vegetables masticated by the molares. In the small intestines, feculent substances are absorbed and saccharified—a fact proved by a simple experiment; fecula taken in the stomach immediately above the pylorus will become blue when placed in contact with iodine, and will, on the contrary, not change color after its passage through the pyloric orifice. It is this, the principal phenomenon of digestion in the duodenum, which has led to the discovery of the saccharifying power of the pancreatic secretion. Hence not only a change in the theory of digestion, but in the pathology of diabetes; we can no longer admit that the kidneys secrete sugar, but that they allow the passage of the saccharine matter contained in the blood.

All these discoveries are in themselves important scientific acquisitions; but their importance is doubled when their practical consequences are reflected upon. The whole history of gastralgia, *rudis indigestaque moles*, must begin anew. No theories can be compared to the recent discovery of the following facts. Eat meat, the urine becomes acid; eat vegetables, it immediately becomes alkaline.

The gastric juice is a powerful acid which readily gives birth by fermentation to gaseous products. In dyspepsia it is therefore a mistaken practice to recommend the use of alkaline salts, by which the digestion of animal food is retarded. The corrosive nature of the fluid accounts for gastric pain, pyrosis, &c., most probably the result of its contact with dry portions of the mucous membrane. By fermentation in the stomach, foul breath and flatulency will be produced; alkaline medicines will be of no avail, but mild laxatives are fully indicated. The digestive power of the gastric juice varies with its heat: below 10 degrees and above 35 degrees, that power diminishes and is completely lost beyond 50 deg. It is therefore not proper to eat very hot substances.

The stomach being the organ in which animal food is dissolved, meat should not be given in gastric affections; whereas feculent substances, digested in the jejunum, can be safely permitted.—*Med. Times* from *Jour. de Méd.—Ibid.*

